# Flatulent Colic in a Horse

**Published:** 1882-07

**Authors:** 


REPORTED BY DRS. WALTON AND M'LELLAN.

COLUMBIA VETERINARY COLLEGE HOSPITAL, NEW YORK CITY, 1882.

---

### IX.—FLATULENT COLIC IN A HORSE.

REPORTED BY DRS. WALTON AND M'LELLAN.

A horse suffering from flatulent colic was brought to the hospital for treatment.

All the ordinary materia medica remedies failing, surgical interference was resorted to. The cereal end of the colon was punctured with a trocar, which gave the animal instant relief.

After the operation, the animal was placed upon a light and fluid diet and kept perfectly quiet for several days.

After five days, he was put to light work.

Ten days after the operation pus commenced to exude from the opening. When the side was examined, it was found that a pocket of pus had developed and burrowed downward ten centimetres.

A counter opening was made at the dependent portion, and a seton introduced. At the end of a week, the wound was perfectly healed.

